# Whole genome sequencing of neurotoxin-producing *Clostridium* species in New York state to bolster epidemiological investigations and reveal patterns of diversity and distribution

**DOI:** 10.3389/fpubh.2025.1651032

**Published:** 2025-11-24

**Authors:** Alexander J. Diaz, Dominick A. Centurioni, Erica Lasek-Nesselquist, Pascal Lapierre, Christina T. Egan, Michael J. Perry

**Affiliations:** 1 Wadsworth Center, New York State Department of Health, Albany, NY, United States; 2 Department Biomedical Sciences, University at Albany, Albany, NY, United States

**Keywords:** botulism, epidemiology, botulinum neurotoxin, whole genome sequencing, bioinformatics, Clostridium, public health

## Abstract

*Clostridia* that produce neurotoxins are highly relevant organisms to public health. While cases of botulism [caused by *C. botulinum* and other organisms that produce botulinum neurotoxin (BoNT)] are rare, the severity of this disease necessitates robust epidemiologic surveillance to promptly identify and mitigate outbreaks. Next generation sequencing (NGS) can provide additional support to these investigations through single nucleotide polymorphism (SNP)-based analysis, phylogenetic reconstruction, toxin subtyping, and structural analysis. Until recently, testing for this disease was restricted to traditional culture or molecular methods such as polymerase chain reaction (PCR) to detect *bont* genes, while mouse bioassay and endopeptidase-mass spectrometry (Endopep-MS) methods confirmed the presence of enzymatically active toxin. The New York State Department of Health (NYSDOH) Wadsworth Center Biodefense Laboratory performed a retrospective whole genome sequence (WGS) analysis of approximately 240 *Clostridium* spp. isolates from the past 40 years to supplement traditional test results and further characterize these organisms. Genomic analyses identified seven BoNT serotypes/serotype combinations, including A4(B5), A5(B2’), and B5F2 that were uncharacteristic of samples typically received. Additionally, SNP-based analysis and *de novo* genome assemblies retrospectively validated several epidemiology links or differentiated samples previously tested with only traditional methods. Our work highlights the clinical utility of supplementing conventional data with NGS to further characterize BoNT-producing organisms and underscores the importance of incorporating WGS into laboratory workflows to support epidemiologic investigations. However, several obstacles still exist which may prevent implementation. These include the expertise needed to execute bioinformatic analyses and interpret the resulting data, a lack of standardized bioinformatic workflows, and difficulty in determining SNP-based thresholds to identify linked samples without incorporation of additional data and analyses. Supplementing or replacing short-read sequencing with long-read sequencing (LRS) and the use of metagenomic or capture-based enrichment for analysis of primary specimens could increase the leverage obtained from WGS in epidemiological investigations.

## 1 Introduction

*Clostridium* species are gram-positive bacteria with the ability to form endospores when subjected to sub-optimal conditions. This results in distinctive central, terminal, or subterminal swellings that allow the organism to persist after exposure to adverse conditions (1). These organisms can be classified into phylogenetically distinct groups/species through various methods such as Amplified Fragment Length Polymorphism (AFLP) analysis, DNA–DNA hybridization, and 16 s rDNA and multigene phylogenies (2–4). *Clostridium* species including *C. botulinum* (Group II), *C. parabotulinum* (Group I), *C. sporogenes* (Group I), *C. novyi sensu lato* (Group III), *C. argentinense* (Group IV), *C. baratii*, and *C. butyricum* produce a potent botulinum neurotoxin (BoNT), the causative agent of botulism (5). Botulism is a relatively rare illness - confirmed in only 243 individuals in the United States for 2021, six of whom were located in New York State (6). Because *Clostridium* spp. can exist under environmentally diverse conditions, such as those found in soils, marine sediments, and the intestinal tracts of animals, the possibility for food contamination is ever present, and often occurs due to the mishandling or under-processing of foods while home canning (7).

BoNTs are encoded by a *bont* toxin gene which, is part of either an *ha+* or *orfx+* neurotoxin gene cluster. These gene clusters are located in several genomic or extrachromosomal locations, based upon the strain and toxin serotype (8), and include genes that encode toxin-associated proteins and a nontoxic, non-hemagglutinin protein as well (3).

BoNTs can be classified into seven serotypes: BoNT/A-/G (9). Of these, BoNT/A, /B, /E, and /F most often affect humans while BoNT/C and /D often affect animals (10). BoNT/G-producing isolates have been recovered from clinical specimens but have not been definitively identified as the cause of illness (11). The different toxin serotypes can be further categorized by the soluble N-ethylmaleimide-sensitive factor attachment protein receptor (SNARE) they cleave. BoNT/B, /D, /F, and /G cleave the vesicle-associated membrane protein (VAMP) SNARE, BoNT/A and /E cleave synaptosomal-associated Protein 25 (SNAP25) and BoNT/C cleaves both SNAP25 and syntaxin (12).

Currently, serotypes are divided into more than 40 subtypes based on amino acid variation (13). Novel subtypes typically display at least 2.6% amino acid variation from presently known subtypes and should form monophyletic groups (3, 14). However, recent studies have shown that inter-subtype variation may not meet these criteria. For example, amino acid variation between serotype B subtypes ranges from 1.6%–7.1%. Some of these subtypes are chimeric or products of inter-subtype recombination and can be found in different genomic backgrounds (3, 15–18), demonstrating the role of horizontal gene transfer and reticulate evolution in shaping Clostridial genomes.

BoNTs and the *Clostridium* spp. organisms that produce them are considered Tier 1 select agents and toxins by the United States Centers for Disease Control and Prevention (CDC) and United States Department of Agriculture (USDA) Federal Select Agent Program, as they “have the potential to pose a severe threat to public, animal or plant health, or to animal or plant products” (19). Toxin activity leads to the acute, symmetric, descending, flaccid paralysis that is characteristic of botulism (20). While treatment with the antitoxin may halt disease progression and prevent death (21), it does nothing to reverse paralysis which often leads to a long recovery with supportive care (22).

Laboratory investigation of botulism cases includes isolation, identification, and characterization of botulinum neurotoxin-producing organisms from clinical specimens and environmental samples. The characterization provided through these investigations is essential considering the potential outcomes of BoNT intoxication, the effect of BoNT diversity on treatment options, and the potential for the deliberate misuse of BoNT. This information assists in determining the source of the exposure and assessing the risk to other individuals in epidemiologically confirmed cases of botulism analysis. Therefore, further characterization of the samples is required due to the impact of BoNT on public health.

Samples submitted to the New York State Department of Health (NYSDOH) for *Clostridium* spp. testing fall into several categories including clinical, clinical surveillance, animal, and environmental. Clinical specimens are those obtained from patients, often including stool and serum, and are used to confirm clinical cases (23). Clinical surveillance samples are those which are believed to have been the cause of illness and often include food or consumer product samples. These are used to determine the source of contamination, conduct outbreak tracing, and assess the risk to the general population. Animal specimens are often collected from carcasses when the cause of death is unknown, but botulism is suspected. These samples are used for sentinel surveillance to detect spillover of BoNT producing *Clostridium* spp. from natural reservoirs into areas which may affect people or livestock. Environmental samples are not directly clinically related but are often sent to the laboratory to assist in epidemiologic outbreak investigations and may include hay, leafy vegetation, soil, dust, and samples collected by law enforcement.

Whole genome sequencing (WGS) using next-generation sequencing (NGS) technologies can assist with investigations and support epidemiological inquiries by providing the resolution necessary to discriminate among closely related isolates. Genomic analyses of *Clostridium* spp. isolates have been performed to compare clinical and environmental isolates related to cases of infant botulism (24), foodborne outbreaks involving nacho cheese (25) or home-canned peas (26), and even wound botulism related to injection drug use (27). Frequently, genomic and phylogenetic analyses strengthen the epidemiological link between patient specimens and outbreak sources. Similar data have been generated for NYSDOH culture collection isolates to retrospectively identify or investigate outbreaks. In this study, the NYSDOH Biodefense laboratory has sequenced approximately 240 *Clostridium* spp. isolates to develop a workflow for the classification of botulinum toxin-producing isolates. Retrospective analysis of this culture collection has identified some rarely observed strains, revealed potentially linked cases, and provided a framework for future analyses that assist in tracing the source of outbreaks in epidemiological investigations.

## 2 Materials and methods

**Figure 1:**
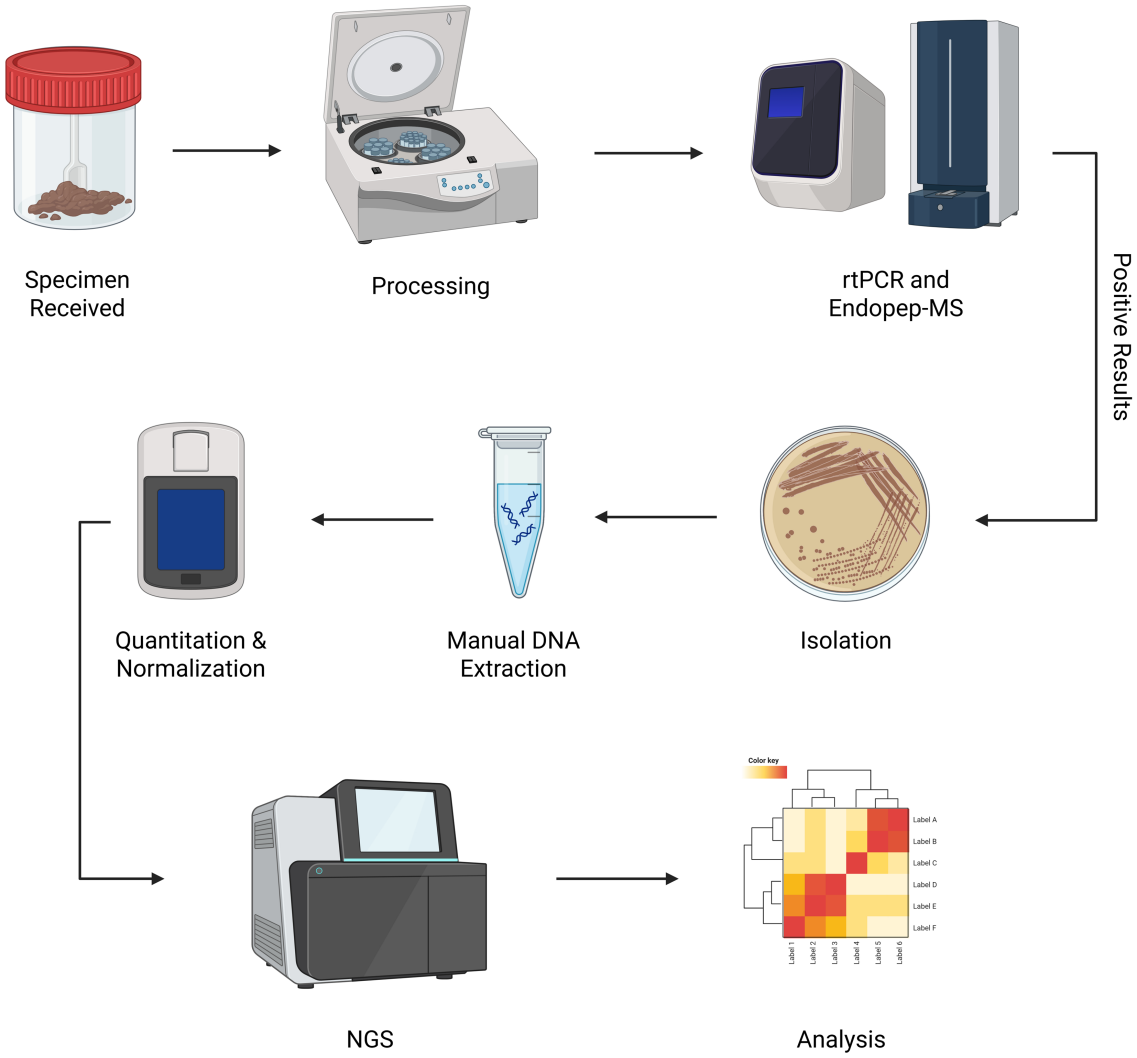
*Clostridium* spp. WGS workflow. Once laboratory testing for confirmation of botulism is approved by epidemiologic investigators, primary specimens received by the NYSDOH Wadsworth Center Biodefense Laboratory are processed and screened for toxin genes using rtPCR. Enzymatic activity is confirmed using Endopep-MS. Isolation of toxin-producing organisms and subsequent WGS is performed to further characterize isolates. Created in BioRender. Centurioni, D. (2025) https://BioRender.com/j4evmmp

No single method is adequate when testing for *Clostridium* spp. Complementary methods must be employed to obtain clinical results as quickly as possible and provide an accurate picture of the situation for diagnostic/epidemiologic concerns. Our laboratory has developed a workflow to optimize this process (Figure 1). By screening suspect botulism samples using an endopeptidase mass spectrometry-based assay **(**Endopep-MS) (28), real-time polymerase chain reaction (rtPCR) (29), and culture (30), positive samples are identified quickly with a high degree of certainty and subsequently sequenced.

Suspect botulism samples tested by our laboratory are processed to generate input material for rtPCR, culture, and Endopep-MS. Samples are further processed to ensure isolation if these conventional methods identify a positive sample. Once isolated, these samples can be batched into groups of 15 to 20 positive specimens to decrease sequencing costs.

### 2.1 Sample processing

For the current study, primary clinical specimens, animal specimens, foods, and other environmental samples including hay, grasses, and leafy vegetation were cut into sections, if necessary, then transferred to filtered stomacher bags. Gelatin diluent buffer (GDB) was added to samples at roughly a 1:1 ratio (g/mL).

As a result of reduced peristalsis associated with botulism, it is often difficult to obtain large volumes of stool for testing. Minimal dilution of these samples is performed, when necessary, to avoid diluting BoNT or organism deoxyribonucleic acid (DNA) below the assay limit of detection but still provide enough volume for testing. Non-pipettable samples were transferred to stomacher bags, homogenized in GDB (2 min, 200 rpm), and the resulting filtrate was used for downstream testing and culture.

Honey samples were processed by diluting 10 g aliquots in 25 mL of phosphate buffered saline. Next, samples were transferred to Nalgene™ Oak Ridge tubes and centrifuged (30 min, 12,000 rpm, 4 °C) using a sealed, fixed angle rotor. Aliquots of the supernatant were retained, and the pellets were resuspended in GDB for use in testing.

Soil samples were prepared by transferring 1 gram of soil to 10 mL of Tryptone Peptone Glucose Yeast Extract (TPGY) broth for enrichment. These samples were incubated anaerobically at 35 °C for 2 to 4 days before testing was performed.

### 2.2 Culture

Enrichment and subsequent isolation of BoNT-producing organisms from primary clinical specimens (excluding serum specimens) and environmental samples was performed before sequencing was attempted. All culture was performed under anaerobic conditions. Approximately 50 μL of processed primary specimen was used to inoculate solid media including Egg Yolk Agar (EYA), Trypticase Soy Agar with 5% Sheep’s Blood Agar (SBA), and *Clostridium botulinum* Selective Medium (CBSM). Approximately 250 μL of processed specimen was used to inoculate TPGY Broth. All culture media was incubated at 35 °C; however, samples that screened positive for the *bont /B, /E,* or */F* toxin gene via rtPCR using the method developed by Davis et al. (29) were also incubated at 25 °C if no growth was observed at 35 °C. EYA and CBSM were checked at 24 to 48 h for lipase or lecithinase-reaction positive colonies, depending on preliminary rtPCR results. Some BoNT-producing organisms do not produce lipase reactions on EYA, which is taken into consideration when no lipase reaction is observed but a positive rtPCR result is generated. After 48 h, TPGY broth is screened for *bont* genes by rtPCR to confirm the presence of viable organism. Positive enrichment broths are then subcultured as described above.

In cases where it was difficult to isolate BoNT-producing organisms from primary clinical specimens or environmental samples, spore selection was performed in two ways. First, 500 μL cell suspensions of suspicious mixed growth or rtPCR positive enrichment broth samples were incubated on a rotary mixer (room temperature, 20 °C–25 °C, 400 rpm,) for 1 hour with 500 μL molecular grade ethanol. Separately, 500 μL cell suspensions of suspicious mixed growth or rtPCR positive enrichment broth samples were incubated at 80 °C for 10 min. Once completed, 25 to 50 μL of each mixture was used to inoculate media directly, while the remaining volume of sample was transferred to TPGY broth for 24 to 48 h, then plated, streaking for isolation.

Due to the Tier 1 select agent designation, use of BoNT-producing strains of *Clostridia* was logged and waste was segregated, destroyed, and disposed of appropriately. Work was performed in a select agent registered Biosafety Level 2 laboratory using appropriate personal protective equipment and enhanced precautions by trained personnel with Department of Justice select agent clearance.

### 2.3 Extraction

Once an axenic culture was obtained, a single, well-isolated colony was transferred into approximately 9 mL of TPGY broth and incubated anaerobically (35 °C) for 24 to 48 h. Cultures were pulse vortexed to obtain a homogenous cell suspension, then transferred to a 15 mL conical tube. Samples were centrifuged using sealed swing-bucket rotors for 10 min, or until a compact cell pellet formed, at 4,000 rpm, 4 °C. Next, all supernatant was removed and destroyed in a 20% bleach solution. Cell pellets were resuspended in 300 μL of a 20 mg/mL lysozyme solution by pipetting, then extracted using a modified Epicentre MasterPure Complete (Lucigen Biosearch Technologies, Hoddesdon, UK) DNA extraction as described by Halpin et al. (31).

### 2.4 Library preparation and sequencing

Library preparation was completed for Illumina sequencing platforms by the Wadsworth Center Advanced Genomic Technologies Cluster. Approximately 100X genome coverage was targeted for each isolate. Sequencing libraries were prepared using the Illumina DNA prep kit with modifications as outlined in Dickinson et al. (32). Samples were run on Illumina MiSeq (v2 500 cycle chemistry kits) or NextSeq instruments (NextSeq 500/550 Mid Output Kit v2.5 300 Cycles).

### 2.5 Genomic analysis

#### 2.5.1 SNP-based analyses

To determine relatedness, each isolate library was compared to a database of 14 *Clostridium* spp. genomes. The reference genome database represents complete high-quality genomes for the following strains: Alaska E43 (CP001078.1), Ba4_657 (CP001083.1), BKT015925 (CP002410.1), BL5262 (GCA_000182605.1), CDC_67071 (CP013242.1), CDC_67190 (CP014148.1), Eklund 17B (CP001056.1), Hall (CP000727.1), Kyoto (CP001581.1), Langeland (CP000728.1), Loch Maree (CP000962.1), Okra (CP000939.1), Osaka05 (NZ_DF384213.1), and Sullivan (CP006905.1) (Supplementary Table S1). The closest matching reference genome for each isolate was identified by MinMash distances using Mash version 2.1.1 (33) and default parameters, except for increasing the number of sketches to 10,000. Species designations were assigned based on the closest matching reference genome (Supplementary Table S1). Isolates lacking a match to the reference database (Mash distance >0.1) were not included in the SNP-based analyses. Reads were filtered with BBDuk from the BBTools suite (version March 24, 2020) (34) and trimmed with Trimmomatic version 0.36 (35) under default parameters. Cleaned reads were mapped to the closest matching reference genome selected in the first step using BWA-MEM Version: 0.7.15-r1142-dirty (36). Alignment files were sorted, and duplicate reads were removed with Picard version 2.9.2-SNAPSHOT (37, 38). Variant positions were required to have a minimum mapping and base quality score of Q20, minimum 10X depth, a quality score (QUAL) > 100, and to be supported by ≥95% of the reads. Sites covered by ≥3X the mean genomic depth were masked to minimize duplicated, repetitive, or unreliable regions in the final consensus genome. Samples with less than 30X average read depth were not included in SNP-based analyses. High quality consensus genomes were generated with SAMTools/BCFtools version 1.4.1 (39, 40) and SNP alignments and matrices were generated using snp-sites 2.5.1 (41) and snp-dists v.0.7.0 (42), respectively.

#### 2.5.2 Toxin serotype/subtype detection by mapping and gene assembly

Toxin serotype and subtype were assigned by mapping reads to a separately curated *bont* subtype database using BWA v.0.7.12-r1039 (36). The database represented full length *bont* genes from each available serotype and subtype deposited in NCBI (Supplementary Table S2). Variants of each subtype were included to maximize the molecular diversity captured. Reads that aligned to any sequence in the database were extracted by Samtools v.1.2-242-g4d5647 (39, 40) and *de novo* assembled with MEGAHIT v.1.2.9 (43). Assembled contigs were then queried against the aforementioned *bont* subtype reference database with BLASTN v.2.13 (44). The top hit was identified and reported if the percent identity and coverage of the reference gene were greater than 70% and 50%, respectively. Cutoffs were set to ensure detection of variable *bont* genes. However, all *bont* genes assembled were highly conserved - with 99%–100% identity to genes from the reference database and 65%–100% coverage (data not shown). Toxin serotype was not reported if blast results could not confidently assign subtype. The presence or absence of *bont* genes was further supported by *de novo* genome assemblies (see below).

#### 2.5.3 *De novo* genome assembly

*De novo* genome assemblies were generated by shovill v.1.1.0 (45) and annotated by Bakta v.1.8.1 (46) using the db-light v.5 database (47). Busco v.5.4.7 (48) determined assembly completeness using the clostridia_odb10.2020-03-06 database (49) and Quast v.5.3.0 (50) provided additional quality metrics. Kraken2 v.2.1.3 (51) assigned taxonomic classification to contigs to estimate assembly contamination levels. Assemblies with <60% Clostridial contigs or Busco scores <90% were not included in *de novo* assembly-based downstream analyses. MOB-suite v.3.0.3 (52) with the mob_recon option detected and classified plasmids, and Orthofinder v.3.0.1b1 (53) assigned proteins to orthogroups (homologous groups of proteins, which includes orthologs and paralogs). Multilocus sequence typing (ST) was performed with mlst v.2.23.0 (54) using the PubMLST database (55). Ribosomal Multilocus Sequence Typing (rST) was performed by the PubMLST webserver (55, 56).

Genes annotated as “*bont*” by Bakta v1.8.1 (46, 47) were extracted and clustered at 100% identity by cdhit-est (57, 58) to retain single representatives of each unique sequence. Unique *bont* genes were aligned with those from the *bont* reference database by mafft v.7.453 (59, 60) and a *bont* genealogy was generated by IQ-TREE2 v2.4.0 (61) under a GTR + F + R3 substitution model with 1,000 ultrafast bootstrap replicates (62) to confirm subtyping assignments initially conferred by the mapping and assembly strategy.

#### 2.5.4 SNP-based phylogenetic reconstruction

SNP-based phylogenetic trees for isolates in the Alaska, CDC_67071, and Hall reference groupings were calculated by IQ-TREE2 v2.4.0 (61) with a TVM + F + ASC + G4 substitution model selected by ModelFinder (63). Branch support was evaluated with 1,000 ultrafast bootstrap replicates (62). The number of positions included in the SNP alignments ranged from 90,750 to 161,391. Polymorphic positions due to recombination were identified and masked by Gubbins v2.3.4 (64) for whole genome alignments of Alaska, CDC_67071, and Hall groupings and all SNPs were extracted by snp-sites (41) to determine the effects of recombination on tree topology. Phylogenies were estimated in IQ-TREE2 (61) under the TVM + F + ASC + G4 substitution model. Topologies between initial trees (those based on all SNP positions) and those built by excluding recombinant positions were compared in IQ-TREE2 with the –rf (Robinson-Foulds) function. Because removing recombinant positions did not affect the overall topology of the Alaska, CDC_67071, or Hall phylogenies (with Robinson-Foulds distances ranging from 0–44) nor the relationships highlighted in the results and discussion sections, we do not report on these findings further and refer to the initial trees herein (SNP alignments and trees with and without recombination are available at doi:10.5061/dryad.2z34tmpzz).

#### 2.5.5 Assembly-based phylogenetic reconstruction

A multigene phylogeny for 225 isolates (including reference genomes from SNP-based analyses) was generated from an alignment of 264 single-copy orthologues as identified by Orthofinder (53). Orthologous sequences were aligned by mafft v.7.453 (59, 60) and concatenated into a supermatrix with an in-house Python3 script. All alignments had complete isolate representation and yielded a supermatrix of 83,069 positions with no more than 10% missing data. IQ-TREE2 v.2.4.0 (61) estimated a maximum likelihood phylogeny under an LG + F + I + R4 model selected by ModelFinder (63) with 1,000 ultrafast bootstrap replicates (62). Additionally, a supertree from 900 protein trees for the same isolates was generated by Astral-Pro v.1.20.3.6 (65), which allows for the inclusion of orthologs and paralogs in species tree estimation by accounting for incomplete lineage sorting and gene duplication and loss. Multisequence alignments were generated by mafft v.7.453 (59, 60) and individual orthogroup trees were reconstructed in IQ-TREE2 v.2.4.0 (61) with the LG + F substitution model. Phylogenies were visualized with the ggtree package (66) for R v.4.4.1 (67) (alignments and trees are available at doi:10.5061/dryad.2z34tmpzz).

## 3 Results and discussion

### 3.1 Summary statistics

Over approximately 40 years, more than 240 specimens were archived at the NYSDOH Wadsworth Center Biodefense Laboratory and recently sequenced. While all samples submitted for testing were suspected to be linked to botulism cases in humans or animals, the laboratory did not play an active role in the selection or acquisition of these samples. Clinical presentation and epidemiologic investigation by submitting organizations were used to justify sample submission for laboratory confirmation. As a member of the CDC Laboratory Response Network, the NYSDOH Wadsworth Center may perform testing for other states in the northeastern portion of the country. After quality control, 220 samples remained for analysis, the majority of which originated from New York (162, 73.6%), but samples from the states of Pennsylvania (1, 0.5%), Connecticut (14, 6.4%), Massachusetts (6, 2.7%), Maine (1, 0.5%), New Hampshire (1, 0.5%), and samples of unknown origin (35, 15.9%) were included as well. Isolates obtained from clinical or clinical surveillance specimens account for 62.7% of samples, while 33.6% were from animals, and 3.2% originated from environmental sources (Supplementary Table S3).

**Figure 2:**
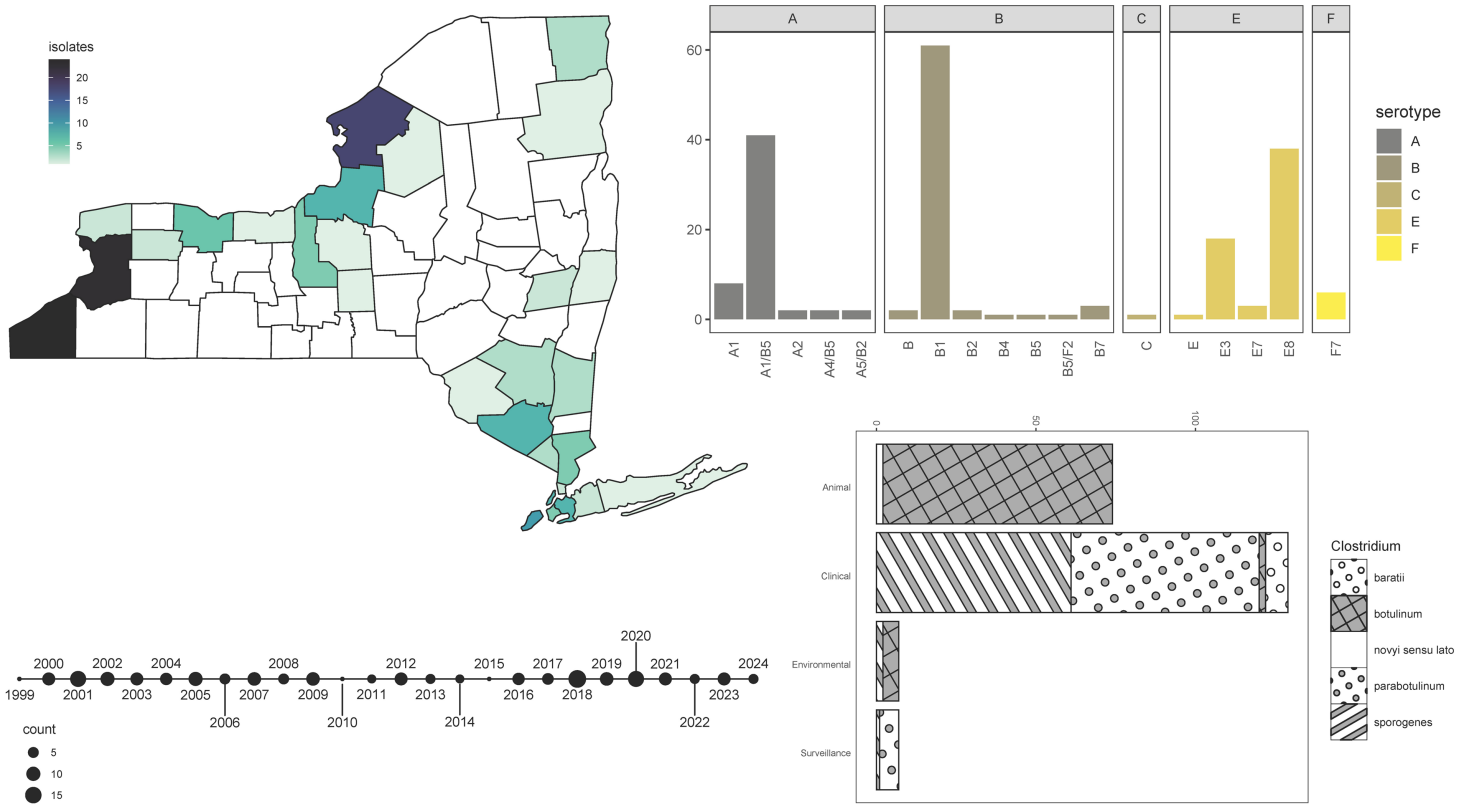
Isolate demographics and characterization. Summary information for 220 *Clostridium* isolates sequenced by the NYSDOH Wadsworth Center. Top left, heatmap, and geographic location for isolates (with available data) collected in New York State; bottom left, timeline reflecting the number of isolates collected for the years spanning 1999–2024 for specimens with collection years available; top right, barplot of the number of BoNT subtypes detected by WGS methods; bottom right, stacked barplot of isolate types and the species they represent with species assignments taken from MASH results. All plots were generated by the ggplot2 (92) package in R (67).

Within our sample set, seven serotype/serotype combinations were observed. Overall, analysis of *bont* genes extracted from WGS data allowed for the designation of 16 BoNT subtypes (Figure 2). The frequency of BoNT types in the collection ranged from as few as one sample producing BoNT/Bf or /C to as many as 70 specimens producing BoNT/B (Figure 2; Supplementary Table S3). All serotype B isolates were designated as Group I proteolytic (*C. sporogenes* or *C. parabotulinum*) except one Group II non-proteolytic (*C. botulinum*) subtype B4 isolate, which derived from an environmental hay sample (Figure 2; Supplementary Table S3). WGS revealed that 41 Group I *C. parabotulinum* genomes harbored *bont/*A1(B5) gene sequences (Figure 2; Supplementary Table S3). These BoNT A(B) isolates produce enzymatically active BoNT/A but not BoNT/B, which is consistent with other reports of silent *bont*/B5 genes in isolates which produce toxin (68). Infrequently observed combinations of *bont* genes included *C. sporogenes* A4(B5) isolates associated with a case of infant botulism in Monroe County, New York), two *C. parabotulinum* A5(B2’) isolates, and one *C. parabotulinum* B5F2 isolate (isolated from another month-old infant from Queens, New York in 2019) (Figure 2; Supplementary Table S3). These rare combination cases appear to conform to previous observations of A4(B5) and B5F2 isolates in cases of very young infant botulism (69). The rare *bont*/B7 gene was detected in three *C. parabotulinum* isolates from clinical stool specimens that differed by 46–126 SNPs (Figure 2; Supplementary Table S3). One BoNT/B7-producing isolate was previously characterized and tied to infant botulism (70). One *Clostridium novyi sensu lato* isolate with a *bont*/C gene was associated with an animal specimen (Figure 2; Supplementary Table S3). Six *C. baratii* isolates collected over a period of at least 16 years harbored the *bont*/F7 gene, which is rarely identified in NYS (Figure 2; Supplementary Table S3). Five of the six F7 producing *C. baratii* isolates were obtained from individuals at least 59 years old (median age 68 years), which likely represent cases of adult toxicoinfections (17). No *C. argentinense* or *C. butyricum* isolates were detected.

### 3.2 *C. parabotulinum* investigations

Most clinical epidemiologically linked isolates were closely related genomically, differing by as little as 0 SNPs; however, as many as 259 SNPs differentiated *Clostridium* spp. isolates obtained from clinical primary specimens and associated clinical surveillance isolates. In one previously reported home-canning incident involving peas (26), *C. parabotulinum* A1(B5) (CDC_67190 reference grouping) was isolated from two clinical stool specimens from different patients, a salad bowl, and an empty jar that contained peas used in the salad. No SNPs were detected between these isolates, reinforcing the epidemiologically identified link (Supplementary Table S4a; Supplementary Figure S1).

Linked strains of *C. parabotulinum* A2, isolated from a primary stool specimen and a commercial honey sample (sourced from a combination of countries including Argentina, the US, and Canada), also showed minimal genomic variation. The clinical stool (IDR2100017857-01-01-2) and honey (IDR2100019300-01-02) isolates differed by just two SNPs but were distinguished from the Kyoto reference genome by 1,545 and 1,647 SNPs, respectively (Supplementary Table S4b). Argentina was previously identified as a reservoir for BoNT/A2 strains (71–73), which underscores the importance of combining traditional epidemiological and WGS data to understand the global transmission and distribution of *Clostridium* spp.

**Figure 3:**
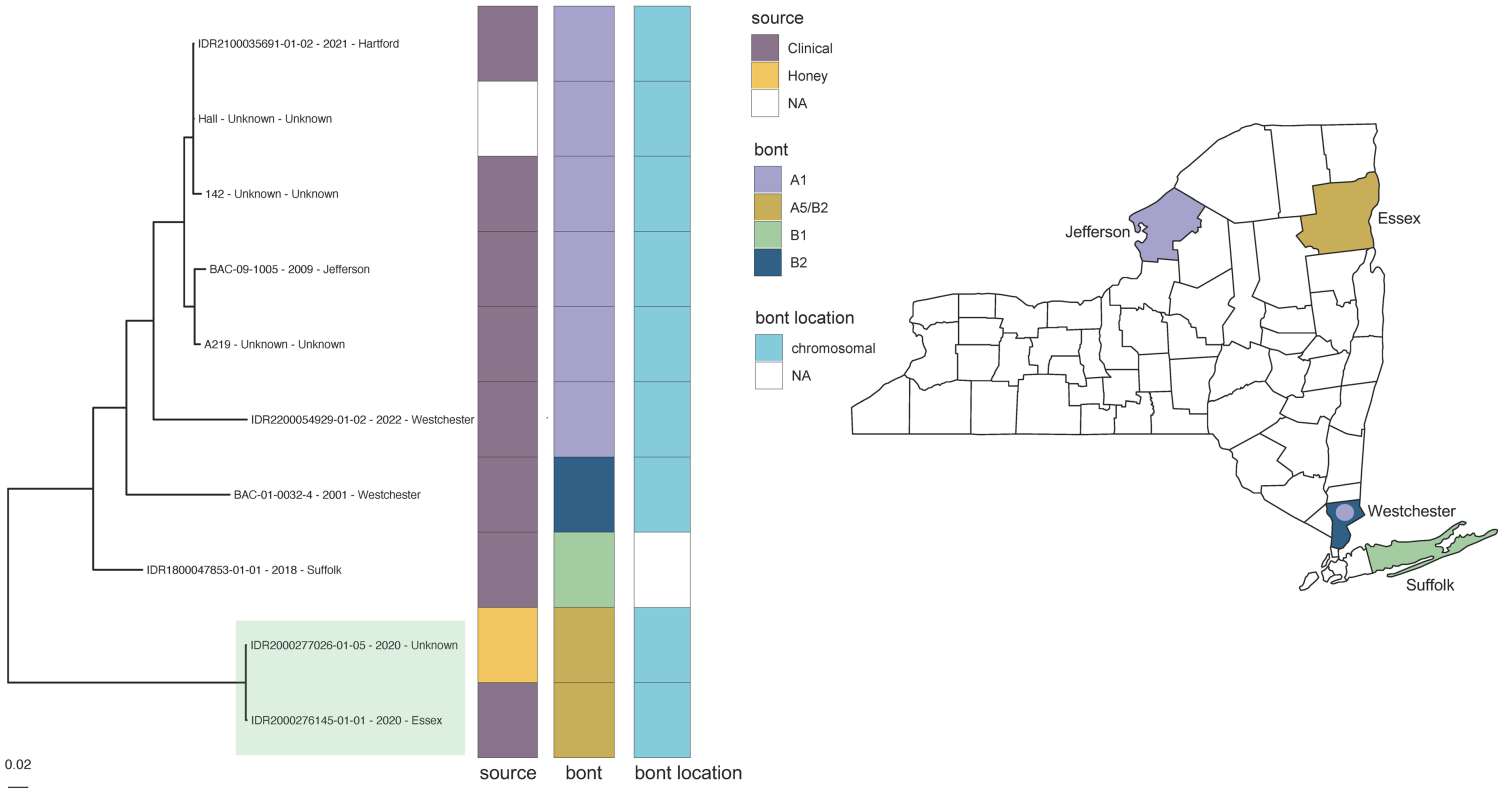
*Clostridium parabotulinum* phylogeny for Hall reference grouping and New York State (NYS) map. Maximum likelihood phylogeny generated by IQ-TREE2 (61) from an alignment of 90,750 SNPs under a TVM + F + ASC + G4 substitution model for *Clostridium parabotulinum* isolates and Hall reference genome. Support values were calculated using 1,000 ultrafast bootstrap replicates (62). Branch support was 100% for all bipartitions. Tips are labeled by isolate name - collection year – county location and are annotated by isolate source, *bont* type, and the predicted location of the *bont* gene. Phylogeny scale bar, substitutions per site. The collection location for NYS isolates with available demographic data are depicted by BoNT type in the NYS map. Epidemiologically linked A5 (B2’) honey and clinical stool isolates (IDR2000277026-01-05 and IDR2000276145-01-01) associated with a case of foodborne botulism are highlighted in green. Trees and associated annotation were visualized with ggtree (66) and maps were created by ggplot2 (92) in R (67).

However, some putatively associated *C. parabotulinum* isolates (Hall reference grouping) displayed higher levels of variation. Epidemiologically linked *C. parabotulinum* A5(B2’) stool and honey isolates (IDR2000276145-01-01 and IDR2000277026-01-05) were differentiated by 259 SNPs in another case of foodborne botulism (Figure 3 green highlight; Supplementary Table S4c). In contrast, the reference Hall genome deviated from a genome sequenced in this study (IDR2100035691-01-02) by just 187 SNPs (Figure 3, Supplementary Table S4c). The increased variation between honey and stool isolates could be due to the presence of polymorphic populations in the original samples (74), but also questions the legitimacy of the link. Incorporating additional A5(B2’) genomes, such as those from closely related isolates predominantly associated with cases of wound botulism in the United Kingdom (69), would clarify the relationship among these isolates. Inspecting variation within a larger context reinforces the conclusion that SNP-based analyses cannot solely determine an epidemiologic link.

**Figure 4:**
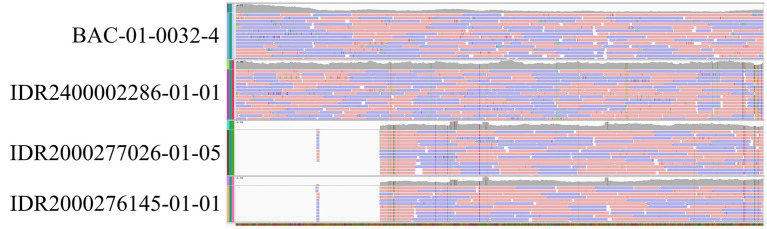
*Bont* B2 gene truncation confirmed by WGS. Illumina paired-end reads mapped to the *bont*/B2 gene from reference strain Su1036 (CP022397.1) for four *C. botulinum* isolates using Integrative Genomics Viewer (93). Isolates BAC-01-0032–4 and IDR2400002286-01-01 did not display a truncated B2 gene, while a large deletion was observed in the epidemiologically related isolates IDR2000277026-01-05 and IDR2000276145-01-01. *De novo* assembly and annotation were also performed, which supported the presence of a deletion.

While WGS identified the toxin subtype for both samples as *bont*/A5(B2’), rtPCR and Endopep-MS results were positive for serotype A only. Further investigation revealed that these isolates contained a large deletion in the *bont*/B2 gene (Figure 4), which is targeted by the rtPCR assay, explaining the negative rtPCR and Endopep-MS results. Isolates harboring truncated, non-functional *bont*/B2 genes in this rare arrangement have been described previously (75), supporting the legitimacy of these partial deletions. Thus, NGS may enhance epidemiological investigations by clarifying discrepant results among methods.

### 3.3 *C. sporogenes* investigations

**Figure 5:**
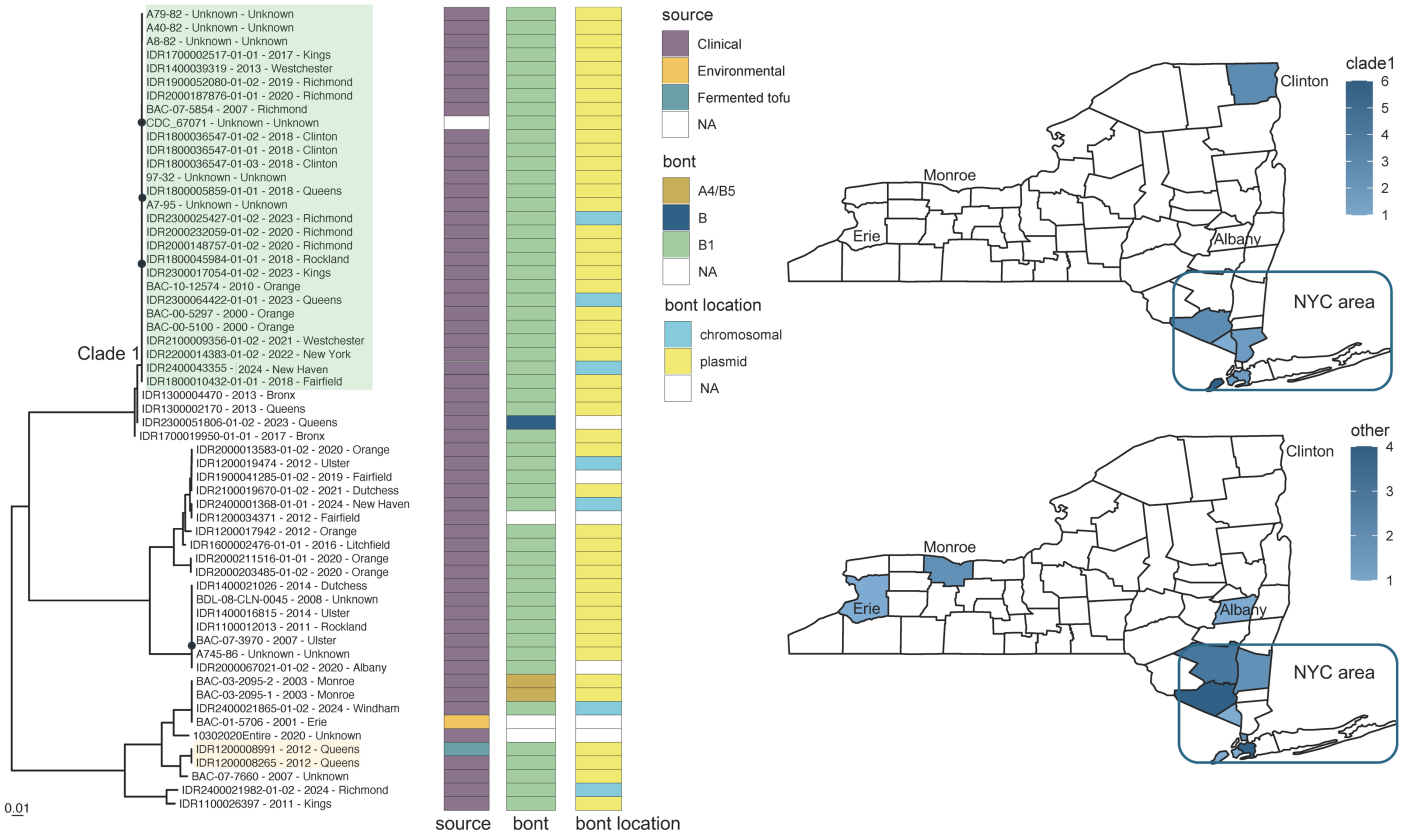
*Clostridium sporogenes* toxin subtype B1 phylogeny for CDC_67071 reference grouping and New York State (NYS) map. Maximum likelihood phylogeny generated by IQ-TREE2 (61) from an alignment of 161,391 SNP positions under a TVM + F + ASC + G4 substitution model for *Clostridium sporogenes* isolates harboring toxin subtype B1 genes and CDC_67071 reference genome. Support values were calculated using 1,000 ultrafast bootstrap replicates (62). Support values <80% are indicated by a black circle. Tips are labeled by isolate name - collection year – county location and are annotated by isolate source, *bont* type, and the predicted location of the *bont* gene. Phylogeny scale bar, substitutions per site. IDR1200008991 and IDR1200008265 associated with a case of foodborne botulism involving tofu are highlighted in light orange. Clade 1 (green highlight) represents a possible clonal expansion in New York City and surrounding metropolitan area. The two NYS maps show the collection locations for clade 1 isolates (top) and all other isolates (bottom) with the color intensity representing the number of isolates. Trees and associated annotation were visualized with ggtree (66) and maps were created by ggplot2 (92) in R (67).

Retrospective WGS also supported investigations involving *C. sporogenes* (CDC_67071 reference grouping) - specifically, a case of botulism associated with the consumption of home-prepared fermented tofu made with ingredients obtained at the local supermarket. Both genomes from the clinical stool and fermented tofu isolates (IDR1200008265 and IDR1200008991) contained the *bont* B1 gene and formed a phylogenetically distinct cluster with identical consensus sequences (Figure 5, orange highlight; Supplementary Table S4d).

Neither genomic variation nor phylogenetic relationships among *C. sporogenes* isolates was correlated with collection date. Variation within one monophyletic group of *C. sporogenes* BoNT/B1 isolates (Figure 5, clade 1 in green highlight), with known collection dates between 2000 and 2024, ranged from 20 to 108 SNPs in comparison to the CDC_67071 reference genome (Supplementary Table S4d). The majority of these isolates could be traced back to New York City (Bronx, Kings, New York, Richmond, and Queens counties), and the surrounding metropolitan area [Westchester, Orange, and Rockland counties in NY and Fairfield County in Connecticut (CT)], when demographic data were available. Comparatively, isolates falling outside of this cluster varied by approximately 1,620 to 77,184 SNPs when mapped to the reference (Figure 5; Supplementary Table S4d). Although no known epidemiological links exist, highly similar monophyletic isolates collected over 20 years in a circumscribed geographic area resembles a pattern of clonal expansion. Clade 1 could be endemic to the region with limited export to other locations (such as Clinton County, NY) or it could represent an importation event of a successful, more widely distributed clone. The distribution and phylogeographic origins of this variant would require a more global representation of genome sequences from *C. sporogenes*, which is beyond the scope of this paper.

### 3.4 *Clostridium novyi sensu lato* investigation

One *Clostridium novyi sensu lato* BoNT/C isolate was obtained from a cow liver specimen (IDR1800052927-02-03) on a dairy farm about 30 miles east of the Lake Ontario shoreline. While BoNT/C is often associated with cases of botulism in waterfowl (76), mammals are also susceptible (77). An animal from the farm or Lake Ontario may have contaminated livestock feed, similar to findings in other recent investigations (78, 79).

### 3.5 *Clostridium botulinum* investigations

**Figure 6:**
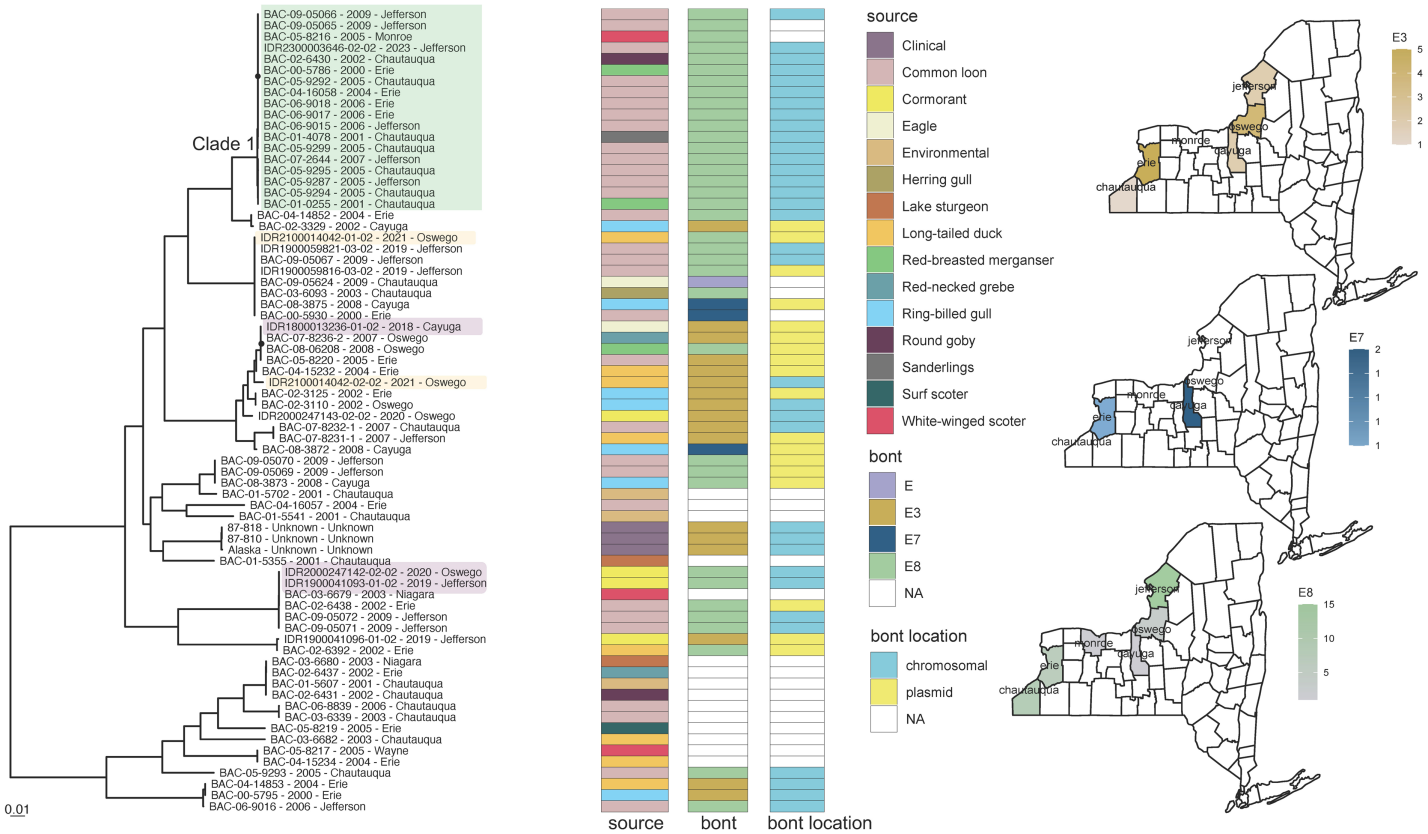
*Clostridium botulinum* toxin serotype E phylogeny Alaska reference grouping and New York State (NYS) map. Mid-point rooted maximum likelihood phylogeny generated by IQ-TREE2 (61) from an alignment of 121,606 SNP positions under a TVM + F + ASC + G4 substitution model for *Clostridium botulinum* serotype E isolates and Alaska reference genome. Support values were calculated using 1,000 ultrafast bootstrap replicates (62). Support values <80% are indicated by a black circle. Tips are labeled by isolate name - collection year – county location and are annotated by isolate source, *bont* type, and the predicted location of the *bont* gene. Isolates from similar geographic areas separated by 6 to 27,600 SNPs (IDR1800013236-01-02, IDR1900041093-01-02 and IDR2000247142-02-02) are highlighted in light purple. Isolates from the same duck with different BoNT subtypes are highlighted in light orange (IDR2100014042-01-02 and IDR2100014042-02-02). Phylogeny scale bar, substitutions per site. The three maps depict the distribution and number of *bont*/e subtypes collected in NYS in this study. Trees and associated annotation were visualized with ggtree (66) and maps were created by ggplot2 (92) in R (67).

Animal isolates offered insight into the evolutionary dynamics of *Clostridium botulinum* serotype E organisms in New York State. *C. botulinum* was isolated from only 1.5% of clinical specimens (2/131) in contrast to 97% of all animal specimens (72/74) sampled (Figure 2), despite clinical specimens being collected from a wider geographic distribution and longer timeframe (Supplementary Table S3). *C. botulinum* isolates derived predominantly from various bird species in New York counties that border Lake Erie and Lake Ontario but also from three fish species and sediment samples. Isolates from different animal species were broadly distributed throughout the phylogeny (Figure 6), supporting a lack of host specificity (15). Two isolates collected from double-crested cormorants on the Lake Ontario shore (IDR1900041093-01-02 and IDR2000247142-02-02) contained *bont*/E8 genes and differed by only 6 SNPs while a third specimen, collected from an eagle only about two miles inland (IDR1800013236-01-02), contained the *bont*/E3 gene and differed from the shore samples by ~27,600 SNPs (Figure 6; Supplementary Table S4e). *C. botulinum* subtype E8 and E3 isolates were also obtained from a single long-tailed duck (Figure 6). Collectively, these results highlight the presence of multiple BoNT/E subtypes in a phylogenetically diverse community of *C. botulinum,* which could contribute to avian botulism in a single geographic region (80). *C. botulinum* outbreaks due to contaminated fish from the Great Lakes (such as Lake Ontario and Lake Erie) have been recorded since the 1960s. Our study suggests that birds may be highly susceptible to infection from fish as well (81).

Some *C. botulinum* serotype E isolates collected decades apart from environmental and animal sources showed limited genomic variation, such as members of a large BoNT/E8 clade (Clade 1, Figure 6) that differed by 0 to 123 SNPs but by approximately 5,500 to 40,000 SNPs compared to other isolates within the Alaska reference grouping (Supplementary Table S4e). These E8 isolates derived from four New York counties spanning approximately 300 miles, all bordering naturally linked Lake Erie and Lake Ontario. The low levels of variation among these geographically constrained, temporally diverse E8 isolates in addition to the chromosomal location of their *bont* genes might reflect stable environmental reservoirs that have facilitated expansion and long-term dormancy, which limits the introduction of mutations via replication (82).

However, several clades across the *C. botulinum* tree were comprised of members with different BoNT subtypes. The presence of different BoNT subtypes in closely related isolates as well as the presence of the same subtype in diverse genomic backgrounds in both chromosomal and plasmid locations support the influence of horizontal gene transfer and recombination in the wide-scale transmission of these genes. Indeed, *bont* E8 itself was determined to be the product of recombination as it shares regions of similarity with subtypes E2, E6, and E7 (15). Group II spores (including *C. botulinum*) are usually less resilient, particularly against temperature (83) and heat (84), with lower optimal growth temperatures than Group I proteolytic organisms. Such factors might contribute to the prevalence of serotype E organisms in the northwestern parts of New York, but lack of environmental and animal sampling from other regions of the state prevents any strong conclusions.

### 3.6 *De novo* assembly results

*De novo* assemblies were 84%–100% complete according to Busco scores with contamination levels ranging from 0%–94%. Mixed or contaminated libraries were also evidenced by duplication level and assembly size (Supplementary Table S3). Most assemblies fell within the standard 2.5–6 MB size range for members of the *Clostridium* genus but some exhibited sizes exceeding 8–9 MB, indicative of genetic material from other sources (Supplementary Table S3). While contaminated assemblies were excluded from downstream analyses (i.e., orthology assignment and phylogenetic reconstruction), mapping-based assemblies distinguished between *Clostridium* and non-*Clostridium* reads, and in some cases, enabled SNP-based analyses to proceed if genome coverage was acceptable. However, the isolates discarded from both analyses were largely consistent with each other (data not shown). As these were clonal isolates, we did not anticipate the contamination levels detected in some of the assemblies. In the future, we would employ a filtering step before *de novo* assembly to avoid data loss.

Analysis of annotated *bont* genes from *de novo* assemblies confirmed the results of the mapping and gene assembly strategy. *De novo* assembly and annotation identified 187 isolates with *bont* genes, which were clustered at 100% identity into 33 groups, reflecting a high degree of sequence conservation within the dataset (Supplementary Table S5). Representative sequences were selected from each group to generate a bont gene tree and evaluate subtyping assignments. With the exception of two sequences (from IDR2100017857-01-01-2 and IDR2100035691-01-02), annotated genes clustered with those from the *bont* reference database according to the subtypes initially assigned by the mapping and gene assembly method (Supplementary Figure S2). Only partial toxin genes were recovered from the *de novo* assemblies of IDR2100017857-01-01-2 and IDR2100035691-01-02. Although blast analyses supported toxin A2 and A1 subtype designations for these genes, both fell on long branches sister to larger E/F or C/D clades due to their truncated nature (Supplementary Figure S2).

For several isolates, no toxin gene was detected by *de novo* genome assembly or by mapping reads to a *bont* gene reference database. This is likely due to the presence of non-toxin producing isolates in our collection, as was also noted in Hannett et al. (80), or loss of *bont*-containing plasmids through subculture. It is also possible that genome assemblies were incomplete, although Busco completeness scores for 93% of *de novo* assemblies lacking *bont* genes ranged from 93%–100% (Supplementary Table S3). In fact, assembling reads extracted from reference-based *bont* alignments appeared more sensitive in detecting *bont* genes and assigning subtypes than *de novo* assembly. The former detected *bont* genes in 15 isolates and assigned subtype designations for 14 [A1(B5), A1, B1, E7, E8] while the annotation from the latter method showed no *bont* genes present (Supplementary Table S3). Moreover, the mapping and gene assembly-based method was able to detect dual toxin isolates that were not recovered by *de novo* assembly.

### 3.7 Supermatrix and supertree phylogenies

**Figure 7:**
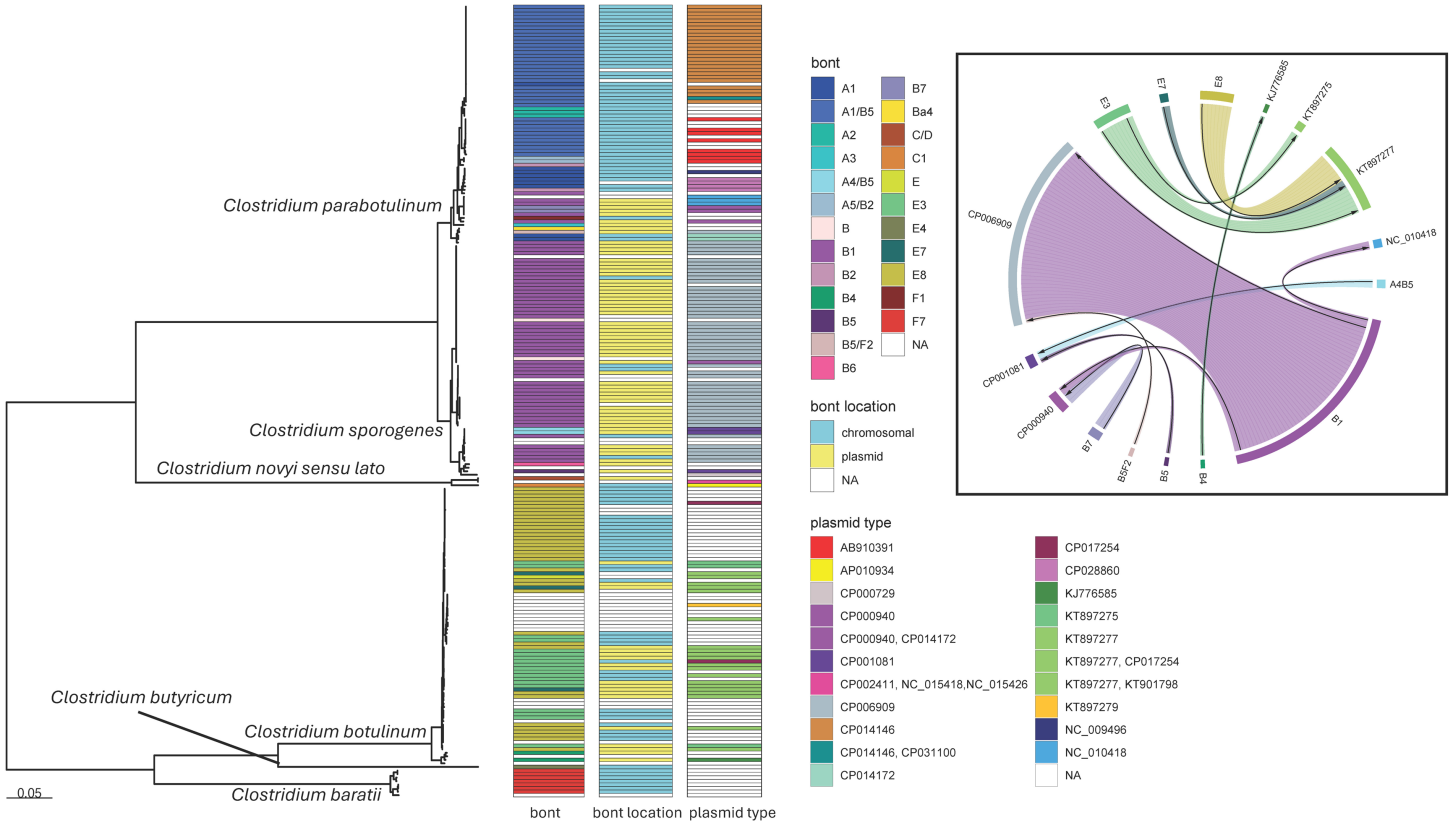
*Clostridium* species maximum likelihood phylogeny and *bont* plasmid distribution. Mid-point rooted maximum likelihood phylogeny of 225 *Clostridium* isolates representing six species. The tree was generated in IQ-TREE2 (61) using an LG + F + R4 substitution model and an 83,069 character supermatrix of 264 single-copy orthologues. Support values were calculated using 1,000 ultrafast bootstrap support replicates (62). Phylogeny scale bar, substitutions per site. All species were recovered with 100% support (not shown). *bont*, *bont* gene serotype or subtype; *bont* location, putative location of the *bont* gene as evaluated by MOB-suite; plasmid type, the GenBank accessions of the closest matching plasmids to those identified in the *de novo* assemblies. Boxed inset, chordogram diagram connecting *bont* gene subtypes to their putative plasmid locations. The direction of the arrow is from toxin subtype to plasmid. Trees and associated annotation were visualized with the ggtree (66) package and the chordogram was generated with the circlize (94) package for R (67).

The topologies recovered by the supermatrix and supertree strategies were roughly equivalent (Robinson-Foulds distance calculated by IQ-TREE2: 292) and both confidently differentiated species into phylogenetically distinct clades (Supplementary Figure S3). Isolates clustered with their respective reference genomes as identified by MASH distances in the SNP-based workflow, except for two isolates assigned to the CDC_67190 reference grouping that clustered with Hall isolates and two Hall isolates that formed a clade with a CDC_67190 isolate (Supplementary Figure S3). Members of the CDC_67190 group formed several distinct clades that were paraphyletic with the Kyoto clade; however, the monophyly of *C. parabotulinum* (which includes CDC_67190, Hall, and Kyoto) was maintained (Supplementary Figure S3). The patterns of orthogroup presence or absence also differentiated isolates by species (Figure 7) and further validated the initial groupings assigned by the SNP-based analyses (Supplementary Figure S4).

Within our dataset, *C. parabotulinum* showed the greatest diversity of serotypes and the greatest mobility of *bont* genes, with some subtypes distributed across multiple *C. parabotulinum* clades (Figure 7). In general, most isolates with the same toxin subtype clustered together [such as the *C. parabotulinum* A1(B5) or *C. sporogenes* B1 clusters] but examples of closely related isolates with different BoNT types appeared throughout the tree (Figure 7). For example, *C. botulinum* subtype E3, E7, and E8 isolates formed a closely related monophyletic group and putatively harbored 6 plasmid types, with E3, E7, and E8 subtypes observed on the same plasmid background (Figure 7). Five *C. botulinum* genomes contained a plasmid (CP017254) first described in *C. taeniosporum* (85). The majority of *C. sporogenes* isolates had *bont* B1 genes with predicted locations on the same plasmid type (Figure 7). However, toxin serotype B genes were predicted on multiple plasmid types with the B1 subtype putatively located on three different plasmids (Figure 7). The plasmid and chromosomal locations for the same *bont* genes, their distribution throughout the tree, and the diversity of plasmids carrying these genes suggests a high degree of genetic mobility for both Group I (*C. parabotulinum* and *C. sporogenes*) and Group II (*C. botulinum*) organisms.

## 4 Conclusion

Our study increases the WGS data available for *Clostridium* spp., particularly for clinical and animal isolates. The combination of WGS for 220 additional *Clostridium* spp. isolates and their associated metadata will provide contextual and reference genomes for future epidemiological investigations and more broadly assist with understanding the evolution, diversity, and global distribution of these organisms.

SNP-based analyses assigned *Clostridium* species to multiple groups determined by MASH distances to a reference genome database. Separating isolates of the same species into different reference groupings ensures that a highly similar reference genome is employed for accurate mapping and SNP detection, which is crucial for the high resolution required in epidemiological investigations. No definitive SNP-based cutoff has been established for linking epidemiological isolates and our results indicate that no single threshold will be appropriate across *Clostridium* species. Although most isolates in related cases differed by very few SNPs, our results uniquely showed that the number of SNPs differentiating unrelated isolates varied by 0 to 4 orders of magnitude. This variation contributes to the challenges faced in determining evolutionary and taxonomic diversity of *Clostridium* spp. (86). Therefore, each reference grouping should be examined independently and phylogenetic relationships along with epidemiological data, should be considered when determining the connection between an isolate and a potential outbreak. Additionally, this approach fails to capture the broader patterns of genomic evolution and phylogeographic distribution within and between species and limits conclusions when potentially linked isolates demonstrate higher levels of variation.

Limited sample collection related to confirmed clinical botulism cases hinders our ability to establish links between samples that may have an unknown, related, local origin. While our dataset substantially expands the genomic representation of *Clostridium* species, we lack sampling from much of the middle and southern regions of New York State. Thus, conclusions regarding transmission, distribution, and endemicity among regions are limited. Increased efforts for active surveillance through in-home and surrounding environmental sample collection would be beneficial to retrospectively identify sources or provide warning for potential outbreaks. Even with an extensive local environmental database, epidemiologic efforts that rely solely on WGS to elucidate sample origins may be confounded by the importation of organisms in various products sourced from around the world. Therefore, a local environmental database has the potential to mislead investigators without contextualizing genomes within a more global framework.

Incorporation of long read sequencing (LRS) may improve *de novo* assembly of genomes and resolve regions, such as repetitive elements, which challenge short read sequencing (SRS) technology (87). Consequently, LRS platforms have been used in combination with SRS platforms to close genomes with high confidence (88). Some *Clostridium* spp. genomes sequenced with Illumina technology contained areas of poor coverage that LRS technology may improve, including the assembly of plasmids, which may harbor *bont* genes. Additionally, LRS platforms may be run directly within biosafety cabinets in secure areas, such as select agent registered spaces. This reduces turnaround time by eliminating the need for inactivation and verification of sterility prior to transfer of extracts to an external sequencing laboratory.

Complex matrices associated with botulism testing can result in poor coverage of target genomes if metagenomic sequencing is performed on primary samples. While multiplexed PCR-based methods have been developed that can detect *bont* genes, toxin gene clusters, or speciate isolates (4, 29), we are unaware of targeted enrichment or amplicon-based NGS assays for detection or characterization of BoNT-producing *Clostridia.* Such assays could provide important information if primary specimens containing no viable organism are received. It is possible that increased output through non-targeted deep sequencing may allow for characterization of low-level pathogens and eventually approach levels of sensitivity similar to rtPCR.

One of the more difficult aspects of sequencing is results interpretation. Bioinformatic training is necessary to carry out and properly interpret the nuances of data analysis, as current pipelines are not standardized across public health institutions and can be complex. Although botulism is a rare disease, we believe it would be beneficial to establish a national or international database and standardized, freely available, version-controlled analysis for BoNT-producing *Clostridium* species. With the availability of numerous tools and associated parameters for each step of the bioinformatic analysis, workflows must be thoroughly evaluated and compared before deciding which would be used in the standardized approach. While this is time consuming, without a standardized method for comparison, changes to pipeline components will alter results and make comparisons between data sets difficult. Previously described WGS analyses include whole genome multilocus sequence typing, toxin gene cluster analysis, and genome-wide average nucleotide identity (27). We envision that a standardized analysis may also take demographic or product information and sequence analysis results into consideration for comparisons and provide outbreak alerts or epidemiological connections. Automatic recognition of novel serotypes or subtypes could also be incorporated, based on established guidelines (13).

Incorporation of WGS into laboratory testing algorithms has allowed us to further characterize isolates in the NYSDOH Wadsworth Center culture collection. Retrospective analysis led to identification of rare subtypes and elucidated challenges in linking potentially related isolates. We believe that incorporation of NGS into public health laboratories will benefit epidemiological investigations to improve public health outcomes.

## Data Availability

Original datasets are available in a publicly accessible repository. Sample sequence data has been submitted to the NCBI Sequence Read Archive (SRA) under BioProject ID (PRJNA1308155). Orthogroup alignments and associated phylogenies are available at: https://doi.org/10.5061/dryad.2z34tmpzz.

## Supplementary material

SUPPLEMENTARY FIGURE S1 Phylogeny of *C. parabotulinum* and CDC_67190 reference genome. Mid-point rooted maximum likelihood phylogeny generated by IQ-TREE2 (61) from an alignment of 97,907 SNP positions under a TVM+F+ASC substitution model. Support values were estimated using 1000 ultrafast bootstrap replicates (62). Isolates related to the cases of botulism involving home-canned peas are highlighted in green. The tree was visualized with FigTree (89).

SUPPLEMENTARY FIGURE S2 Bont gene tree. A maximum likelihood tree of representative *bont* genes from *de novo* assemblies and *bont* genes from the reference database was generated by IQ-TREE2 (61) under a GTR+F+R3 substitution model with 1000 ultrafast bootstrap replicates (62). Genes from *de novo* assemblies represent unique sequences within the Wadsworth dataset and are labeled by isolateName_geneNumber (colored text). Toxin genes from the reference database are labeled by subtype_AccessionInformation (black text). Subtype clades containing genes from Wadsworth isolates are highlighted. Nodes are labeled with bootstrap support values. Scale bar, substitutions per site. All Wadsworth *bont* genes cluster by the subtypes initially assigned with the mapping and gene assembly method except two truncated sequences (gray text).

SUPPLEMENTARY FIGURE S3 *Clostridium* species supermatrix and supertree phylogenies. A multigene phylogeny for 225 isolates was generated from an alignment of 264 single-copy orthologues by IQ-TREE2 (61) under an LG+F+I+R4 substitution model. A supertree from 900 protein trees for the same isolates was generated by Astral-Pro. Trees were visualized in R (67) with the ggtree (66) package. Tips are colored by the species assigned by MASH distances from the SNP-based analysis. The tip points for isolates in the CDC_67190, Hall, and Kyoto reference groupings assigned by the SNP-based analysis are colored to highlight their paraphyletic nature.

SUPPLEMENTARY FIGURE S4 Heatmap of orthogroup presence / absence data for *Clostridium* isolates. Isolates (columns) clustered by the presence (black) and absence (white) of orthogroups (rows). The heatmap was generated by the ComplexHeatmap (90, 91) package in R (67). The distinct patterns of orthogroups reveals good agreement with species designations assigned by SNP-based analyses.
