# Supplementary figures and images for: Whole genome sequencing of neurotoxin-producing Clostridium species in New York state to bolster epidemiological investigations and reveal patterns of diversity and distribution
Source: Front Public Health. 2025 Nov 24;13:1651032. doi: 10.3389/fpubh.2025.1651032 (PMC12682877; doi:10.3389/fpubh.2025.1651032)

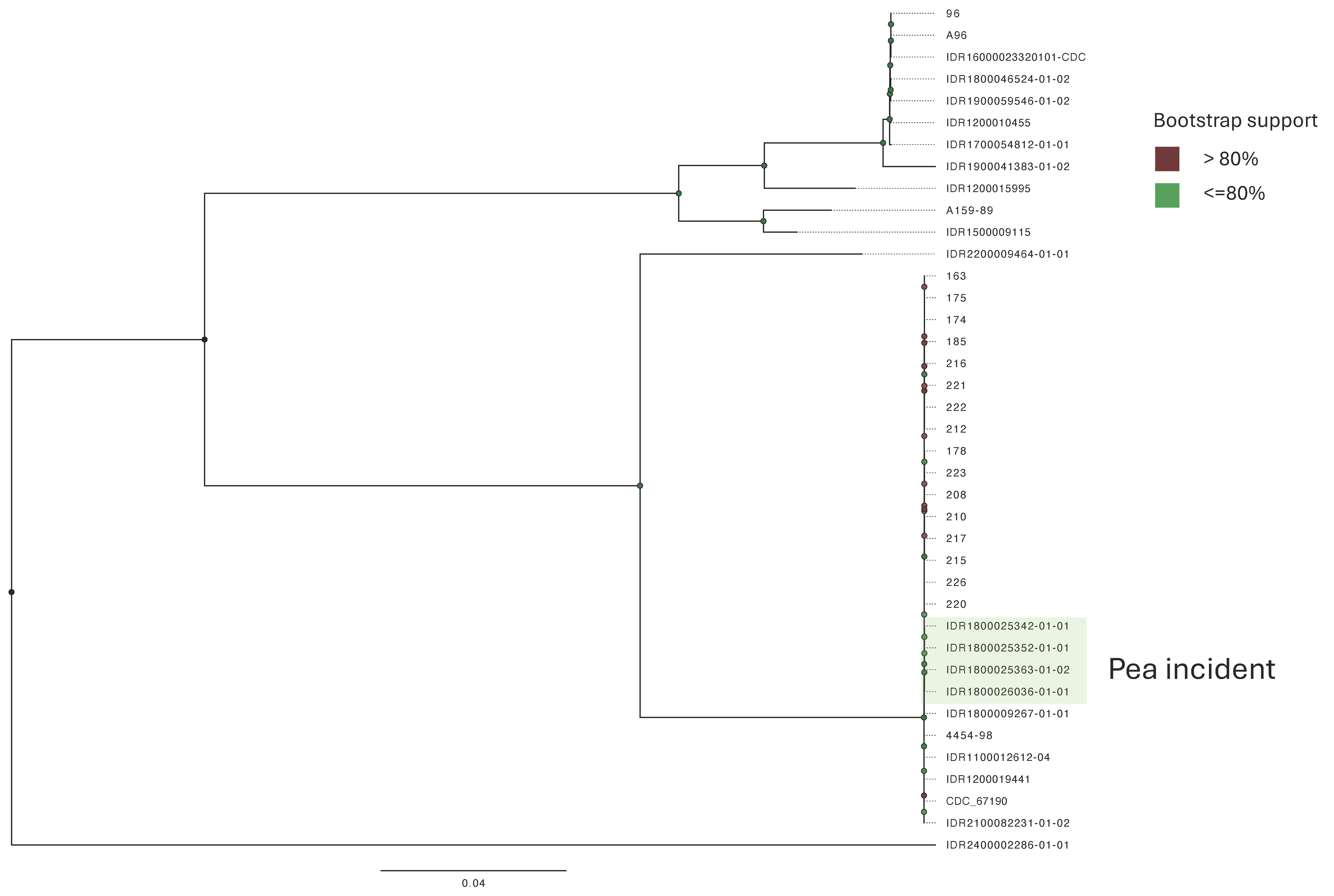

Supplement: Supplementary file 1 [file Image_1.jpeg]

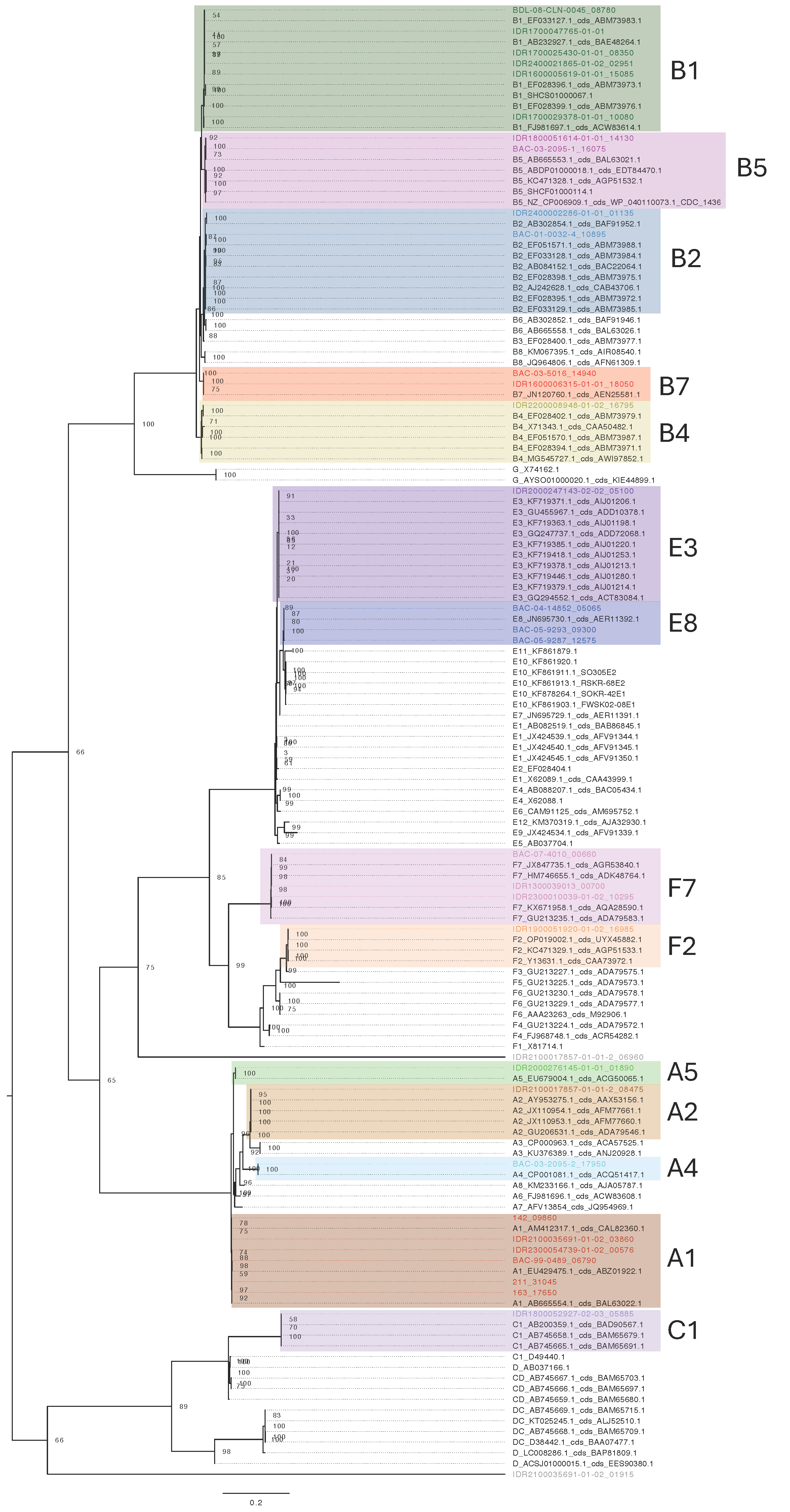

Supplement: Supplementary file 2 [file Image_2.jpeg]

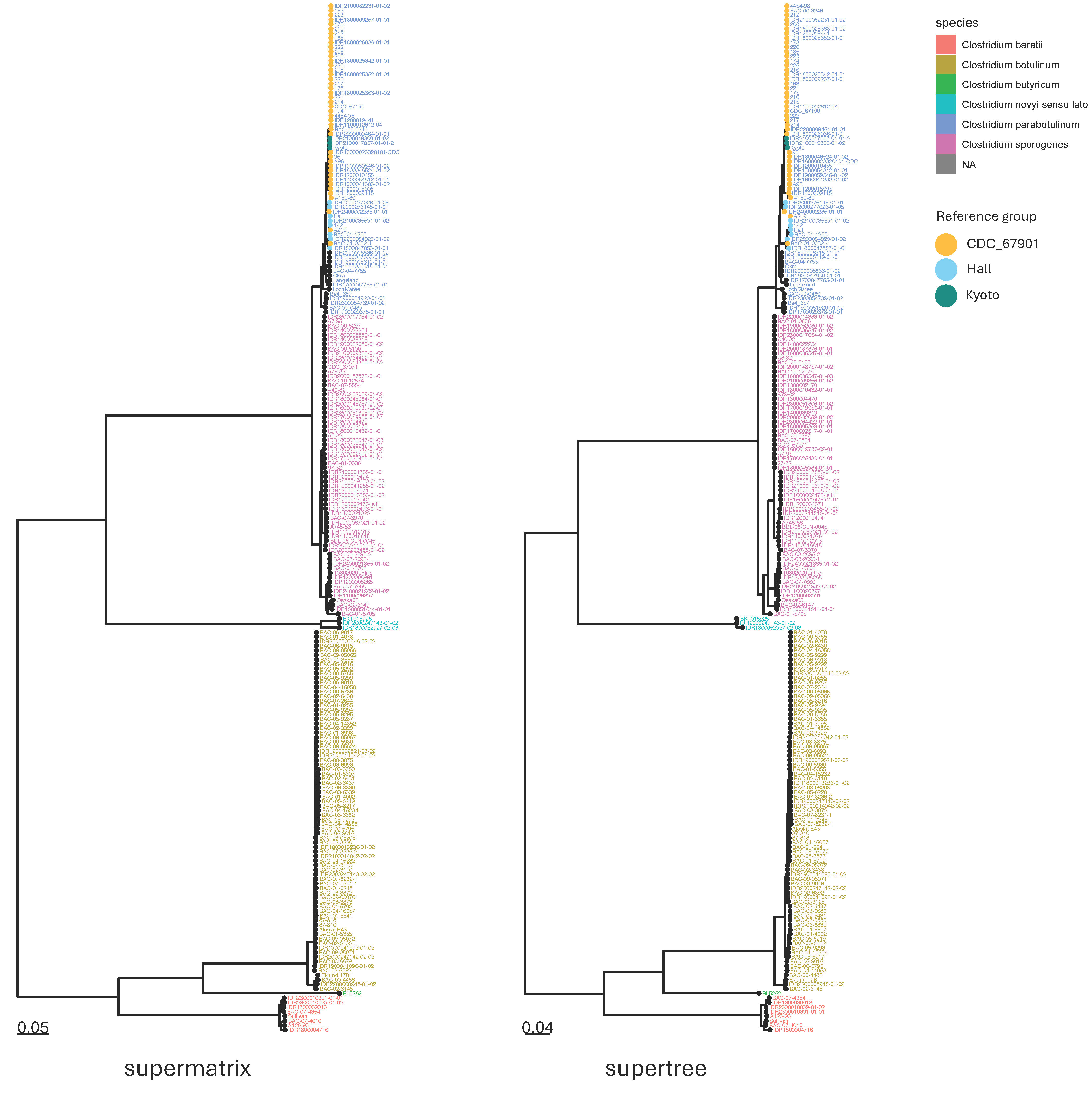

Supplement: Supplementary file 3 [file Image_3.jpeg]

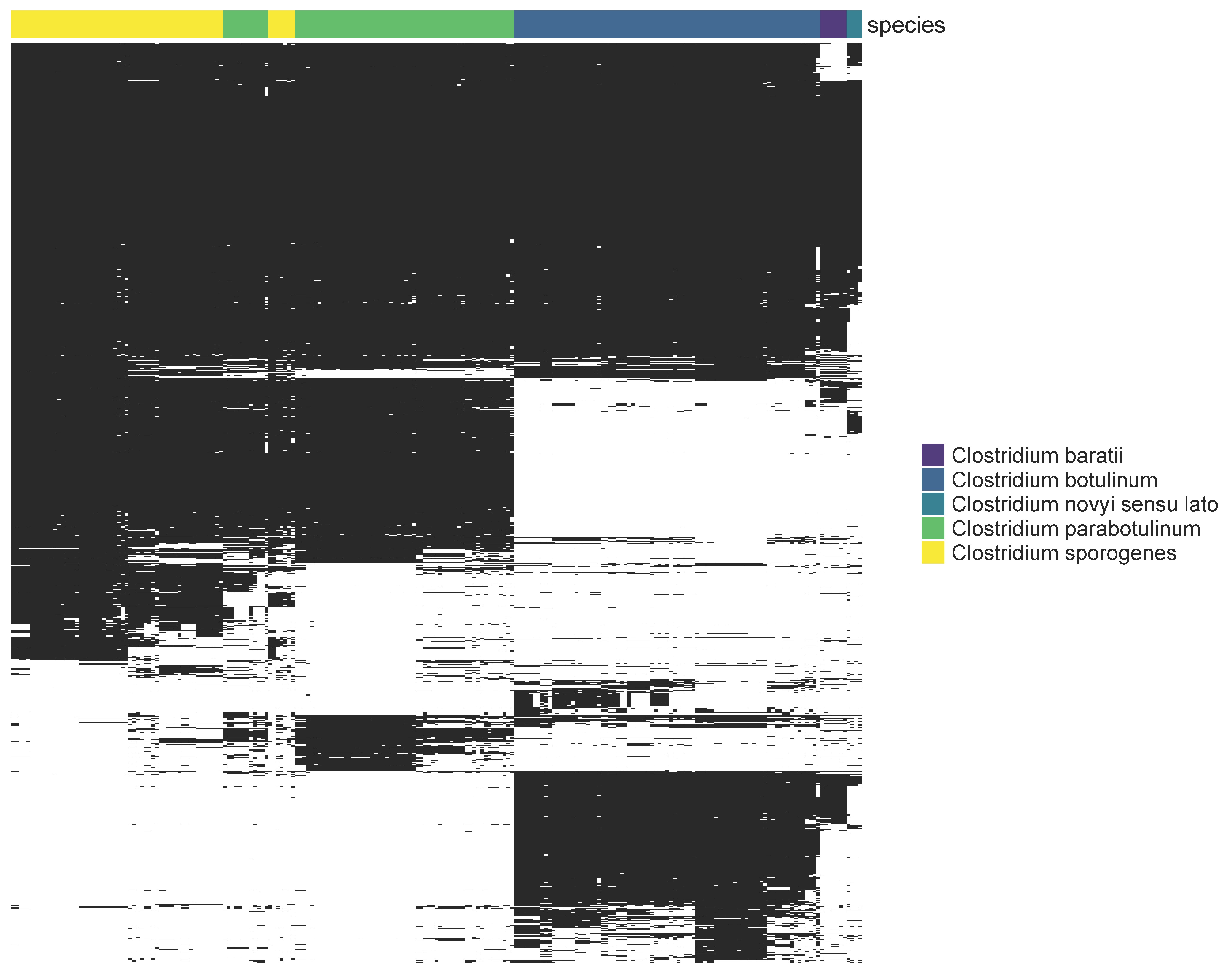

Supplement: Supplementary file 4 [file Image_4.jpeg]
